# Features of and preventive measures against hypertension in the young

**DOI:** 10.1038/s41440-019-0229-3

**Published:** 2019-03-20

**Authors:** Hiroshi Kawabe, Tatsuhiko Azegami, Ayano Takeda, Takeshi Kanda, Ikuo Saito, Takao Saruta, Hiroshi Hirose

**Affiliations:** 1 Department of Internal Medicine, Keio University School of Medicine, Tokyo, Japan; 2 Health Center, Keio University, Kanagawa, Japan

**Keywords:** Blood pressure measurement, Lifestyle, Low birth weight, Obesity, Young hypertension

## Abstract

The Japanese hypertension guidelines report that essential hypertension is detected in 1–3% of upper elementary and high school students during blood pressure (BP) screenings. Hypertension in these age groups is an emerging public health concern mainly attributed to the rising rate of pediatric obesity. Considering the existence of BP tracking phenomenon, early preventive education and instruction are necessary, especially for male students with moderately elevated BP showing a tendency toward obesity, despite the low prevalence of hypertension in high school students. Students with a positive family history of hypertension and those born with low birth weight need the same measures. Lifestyle habits, such as increased alcohol intake, dramatically change once students begin university; thus, early education and instruction regarding the factors influencing BP are necessary. In particular, for male students with higher BP during high school, caution regarding increased body weight is required irrespective of their level of obesity. Young adults aged <40 years should be educated about the association between body weight and hypertension. Particular caution surrounding lifestyle habits, including drinking and smoking, is warranted in male hypertensive subjects because hypertension at a young age is strongly associated with obesity. BP monitoring and the management of obesity should be considered efficient approaches to the detection and treatment of hypertension. For the lifetime prevention of hypertension, it is essential to be aware of one’s health status and learn about healthy lifestyles beginning in childhood. BP measurement may be an appropriate means to achieve this goal.

## Introduction

Considering the existence of tracking phenomenon of hypertension [1–4], preventive measures beginning at a very young age are necessary, as early detection and proper treatment of early-onset hypertension are becoming important concerns. However, with regard to the available knowledge of the incidence of hypertension in children and the elderly, there is very little data about young hypertension in Japan. Indeed, blood pressure (BP) evaluation is not included among the requirements for school medical checkups in the School Health Safety Law in Japan. Naturally, there is almost no concern regarding BP among young students, and the present status or the natural history of young hypertension cannot be easily defined. Moreover, in Japan, it becomes difficult for the educational system to follow a child from an elementary school to a junior high school to a high school and finally to a university. Our private school system of Keio Gijuku provides consistent education from elementary school and has an organization that can follow a child until they become a university student.

In this review, we focused on current hypertension status among Japanese youth and the effective preventive measures against young hypertension, mainly by examining the studies that have followed youth from high school to university over many years in our facilities.

## Definitions and diagnostic criteria of hypertension in youth

Although chapters regarding hypertension in children and hypertension in the elderly are published in “The Japanese Society of Hypertension Guidelines for the Management of Hypertension (JSH 2014)” [5], a chapter regarding hypertension in adolescents and youth does not exist. That is, hypertension in youth remains undescribed, in contrast to hypertension in children or the elderly, and is included under the same category as adult essential hypertension or secondary hypertension. Furthermore, hypertension in youth has never been attributed to a specific pathophysiology.

The definition determining the age range of young hypertension is very ambiguous. Even a 15-year-old (a junior high school student) is usually considered a child, and the period between adolescence and 18 years of age is considered the age between childhood and youth. In the JSH 2014 [5], hypertension in high school students is partly discussed in the chapter relative to hypertension in children. Since Japanese doctors of internal medicine usually target a patient population that includes high school students, we also focus on a population beyond high school students as a target of research. Similarly, an upper limit of age is not clear, and hypertension in individuals below age 35 or 40 is usually classified as young hypertension in many cases. Thus, our research mainly focused on high school students to young adults below the age of 40 years.

The diagnostic criteria for young hypertension in high school students are usually set at 140 mm Hg or more for systolic BP (SBP) or 85 mm Hg or more for diastolic BP (DBP) [5]. For university students (aged 18 or older), a SBP of 140 mm Hg or more or a DBP above 90 mm Hg is defined as hypertension [5].

## Prevalence of hypertension in the young

In the United States, the administration of periodic BP measurements beginning at age 3 is recommended during a general pediatric medical examination [6, 7]. However, BP measurement is not included in the requirements for medical checkups for older school children in Japan. Therefore, in contrast to the accumulated data in the United States, sufficient data have not been accumulated in Japan that can be used to elucidate clear, normal BP percentile values classified by sex, age, and height percentiles.

In the JSH 2014 [5], essential hypertension has been reported to occur in 1–3% of elementary school students in higher grades and high school students upon BP screening [8, 9]. From data for the most recent 3 years (from 2015 to 2017) at our facilities, 0.6–1.8% and 0.5–0.7% of high school male and female students and 3.2–3.7% and 0.2–0.4% of university male and female students, respectively, were classified as having hypertension. There were very few hypertensive women at these ages. With respect to the prevalence of hypertension seen in young adults beginning after graduation from a university to age 40, the National Health and the Nutrition survey in the 2014 fiscal year showed that hypertension was detected in 22.7% of men and 3.6% of women aged 30–39 years and in 10.9% of men and 1.1% of women aged 20–29 years. In an examination of the most recent 3 years in our facilities, 7.5–8.9% of men and 0.8–1.0% of the female faculty and staff aged below 40 years were classified as having hypertension. Thus, it is also clear that there are few hypertensive women in this age group.

The impact of the childhood obesity epidemic on the prevalence of hypertension in younger generations can be seen in several studies from the Houston Screening Project [10–12]. In multiple publications, these investigators have demonstrated an increased prevalence of hypertension among obese children—as high as 4.5%—than among nonobese children. Indeed, a recent examination of BP data in 8- to 17-year-old children from the National Health and Nutrition Examination Survey and other related population-based studies conducted in the United States from 1963 to 2002 clearly demonstrates an increase in the prevalence of elevated BP in children, with much of the increase attributable to the increase in childhood obesity [13]. Based on this analysis, the prevalence of hypertension has reached nearly 4%. Similar findings have been observed in screening studies performed in other countries including China [14] and Iceland [15]. Thus, it is apparent that the increased prevalence of hypertension in children is a global phenomenon, likely related to the worldwide increase in the prevalence of childhood obesity [16]. In this context, the prevalence of obesity (body mass index [BMI] ≥25), which is strongly related to young hypertension, was as follows in our facilities in the past 3 years: 6.3–7.0% and 1.3–1.6% in high school male and female students, respectively, and 10.8–11.0% and 3.0–3.3% in university male and female students, respectively. Moreover, data from our facilities indicate that 16.9–18.2% of the male or 4.4–5.3% of the female faculty and staff aged below 40 years were classified as obese.

Furthermore, it seems that BP in these age groups tends to be influenced by the measurement environment more than that in adults. Therefore, two or more repeated measurements on one occasion are usually recommended for younger individuals. Previous screening programs have demonstrated the importance of performing repeated measurements of BP before labeling an adolescent as hypertensive because studies that have used just one BP determination have found a significantly higher prevalence of hypertension than studies in which repeated measurements were obtained [14].

## BP measurements in young individuals

Although the prevalence of hypertension increases as age increases, it is thought that the origin of the onset of hypertension in adults stems from childhood (tracking phenomenon). Therefore, BP should be monitored as much as possible during childhood, and it seems to be important to assess BP values. However, the opportunity to actually measure a child’s BP is restricted, and currently, even basic data for determining normal values have not been sufficiently accumulated. In Japan, the requirements associated with BP in medical checkups at schools include a medical examination, a urine test, and an electrocardiography test, among others, while the BP measurement itself is not included. Unlike adults, the following points are potential reasons why a child’s BP measurement is rarely performed as a routine part of medical examinations in pediatric subjects or for school hygiene requirements in Japan: (1) unlike adults, hypertension itself is rarely considered in children; (2) for children, the consultation room is a place where anxiety becomes prevalent, and thus, it is not suitable for BP measurement; (3) it is necessary to change the width of the cuff according to the child’s physique; and (4) since normal values differ by sex, age, and height, BP in childhood is difficult to evaluate.

Conversely, the American Academy of Pediatrics (AAP) published new guidelines for pediatric hypertension in 2017 [6]. The clear percentile values of BP classified by sex, age, and height percentile are similar to the table reported in the “Fourth Report on the Diagnosis, Evaluation, and Treatment of High Blood Pressure in Children and Adolescents” published in 2004 [7]. These 2017 AAP guidelines for pediatric hypertension place considerable emphasis on BP measurements, which are essential for the accurate diagnosis of hypertension as well as for treatment titration. A strong commitment of the AAP to obtain more accurate BP measurements in clinical practice is expected to improve pediatric care because, as is the case for adults, BP measurements are the sole test for hypertension diagnosis and management in children.

Many children are over- or underdiagnosed when only office BP measurements (including measurements at the time of medical checkup) are used. Therefore, out-of-office BP evaluations using ambulatory BP monitoring (ABPM) [17] or home BP measurement [18] are often necessary for accurate diagnosis. Although ABPM has received greater attention for children than home BP measurement, it is not easily accessible in primary care or schools, and home BP measurement might be preferred by subjects for repeated evaluations. Therefore, home BP measurement has become more widely available in clinical practice and is used in the evaluation of children with elevated BP. Recently, Stergiou et al. [19] proposed that more research on home BP monitoring in children is urgently needed.

Japanese guidelines for pediatric hypertension have indicated that BP measurement should be repeated three times and that the third value should be adopted as the value for measurement at that time. Furthermore, to diagnose hypertension in children, BP should be measured at least three times on different occasions [20]. During repeated measurements, stress and anxiety at the time of measurement usually disappear, and children’s actual BP value will be approached. In Japan, the original diagnostic criteria for pediatric hypertension were established considering differences in lifestyle, eating habits, environment, physique, and race, among other relevant variables. However, there are few reports available on children’s BP to serve as the basis for comparison. In the future, a medical checkup at school may serve as an ideal place to facilitate wider accessibility of children’s BP measurement in Japan. Moreover, households having an electronic semiautomated sphygmomanometer are increasing in number given the progression of lifestyle-related disease prevention. Therefore, it is expected that the performance of BP measurements for all family members at home will provide families with an opportunity to learn about the current health conditions and to become aware of the risk of future cardiovascular events. The authors believe home BP measurement will serve as an aid to health education.

## Influence of genetic factors

The hereditability of BP was established decades ago by identifying a correlation between the BP levels of parents and their natural offspring but no correlation between parents and those of adopted children [21–23]. Thereafter, several published studies have demonstrated that a large percentage of children and adolescents with essential hypertension have positive family histories of hypertension in parents or grandparents [24, 25]. Genetic influences on BP have also been shown frequently in comparison studies of siblings [26] and twins [27].

Thus, genetic factors, along with environmental factors, play an important role in the pathogenesis of hypertension. It has been reported that individuals below the age of 50 years with a family history of hypertension have a fourfold risk of developing hypertension compared with individuals without a family history of hypertension [28]. Because early detection and treatment of abnormal physiological conditions associated with hypertension can prevent or postpone end-organ damage or a morbid event, it is important to identify individuals at high risk before hypertension develops.

We previously investigated the relationship between home BP and body weight in young normotensive men (mean age 16 years) with normotensive parents and age- and sex-matched normotensive men with one or both parents being hypertensive [29]. In this study, our subjects were high school students, and their mothers were between the ages of 40 and 50 years. Because women in this age group were unlikely to have measured their own BP, hypertension may have been unrecognized in some of them. Furthermore, although a family history of hypertension can be determined from histories or questionnaires obtained from parents and grandparents of subjects, a diagnosis of hypertension should be based on BP measurements. Since casual measurements of BP have been found to be an unreliable method for evaluating hypertension [30–32], we asked parents as well as subjects to measure home BP with an electronic semiautomatic sphygmomanometer. We determined the family history of hypertension on the basis of home BP measurements.

In our previous study, students with positive family histories of hypertension had higher body weight than students without a family history of hypertension [29]. Although the BP values at the time of medical checkup were similar between normotensive high school students with hypertensive parents and those with normotensive parents, home SBP was significantly higher in students with hypertensive parents than in students with normotensive parents, despite SBPs being within the normal range [29]. Furthermore, significant positive correlations between body weight or BMI and home SBP were observed only in students with a family history of hypertension [29]. These results indicated that young normotensive subjects with a genetic predisposition to hypertension weighed more and had higher home SBPs than subjects without a family history of hypertension. Our observations further indicated a close relationship between a family history of hypertension and increased body weight, even in young normotensive men. Therefore, students with a positive family history of hypertension need to pay specific attention to increases in body weight during high school, as the possibility that this awareness may lead to the prevention of or delay in the development of hypertension has been suggested. To check for predisposition, it is necessary to educate individuals as early as possible so that all family members may measure home BP periodically.

It is possible that a number of subjects with hypertensive parents also had a positive family history of being overweight [33]. We can offer no pathophysiological explanation for the increase in body weight observed in prehypertensive subjects with family histories of hypertension. However, obesity-induced hypertension is a common pathway observed in both children and adults [34–36] and an updated comprehensive overview of the pathogenetic factors and pathophysiological mechanisms linking obesity to hypertension has recently been reported [37].

## Influence of low birth weight and early childhood growth

In Japan, the mean birth weight has been decreasing since the 1980s [38], and the population born with low birth weight has been increasing, which is similar to the observations made in many other developed countries [39, 40]. According to the Vital Statistics of Japan by the Ministry of Health Labor and Welfare, the mean birth weight decreased from 3.20 kg in 1980 to 3.02 kg in 2009. In contrast, the percentage of low-birth-weight infants (<2500 g, single delivery) increased from 4.6% in 1980 to 8.3% in 2009. Several factors, such as shorter gestational duration, increased maternal age, parental socioeconomic status, and multiple fertilizations, have been reported to affect birth weight. Low prepregnancy BMI, due to dieting, and low maternal weight gain, due to strict weight management, also seem to be major contributors to the high incidence of low-birth-weight births in Japan [41].

The concept of early nutrition, as reflected in anthropometric variables measured at birth and during infancy, as an important contributor to elevated BP has received substantial attention in the medical literature [42], particularly from a group of investigators from the United Kingdom. Barker [43] showed that men and women whose birth weights were at the lower end of the normal range, who were thin or short at birth, or who were small in relation to placental size had increased rates of hypertension and coronary heart disease. This low-birth-weight hypothesis has received considerable support from the growing evidence that BP in adult life is inversely related to birth weight [44]. Furthermore, population studies conducted by Barker and others have demonstrated an inverse correlation between birth weight and adult BP [45–47]. This observation, however, has not been confirmed by other studies [48], and Paneth et al. [49] objected to this low-birth-weight hypothesis because potential confounding variables, especially social class, had generally not been taken into account. Furthermore, since the relationship may not be demonstrable during childhood or early life [50], the data concerning birth weight and BP during adolescence are thus far inconsistent and controversial [51].

According to previous results in Japan, BP at the age of 3 years was higher with decreasing birth weight and increasing body weight [52]. Moreover, in a 20-year follow-up of 4626 individuals from birth, a lower birth weight and a smaller rate of increase in height from age 3 to 20 years were independently associated with, respectively, increases in BP and serum cholesterol levels at the age of 20 years [53]. In adolescence, however, when the tracking of BP is perturbed by rapid growth, no consistent association was apparent [50, 51, 54]. In our previous study, we could not find any significant relationship between birth weight and BP in either male or female high school students (mean age 15 years) [55]. However, a significant relationship between body weight and BP was observed in men but not in women [55]. A stepwise regression analysis performed using body weight, the body weight/birth weight ratio, BMI, and the BMI/birth weight ratio identified birth weight as the independent variable. Although BMI was associated with both SBP and DBP in male subjects, this association was not observed in female subjects. These results were in line with those reported by Siewert-Delle et al. [56], who showed that adult BP was not associated with birth weight but only with adult BMI in male subjects born at term or later. Similar results had previously been reported in the Muscatine Study [57] and in a paper by Holland et al. [58], who reported that weight gain in early childhood increased the risk of higher adult BP, particularly in men.

These results indicated that the influence of BMI was greater than that of birth weight for BP in men of this generation. However, we do not know which of the three candidate factors (low birth weight, overweight, and weight change) was the most important factor determining adult BP. The authors deem it important to determine which factor or combination among the three candidate factors will contribute to the onset of hypertension in adults. Conversely, the influence of BMI was not observed in women. The following may be involved in this sex difference: (1) young women rarely exhibit hypertension; (2) the range of BP levels is narrow; and (3) these women are also rarely overweight. Therefore, if a larger sample is examined, the same tendency that was observed in men may also be observed in women. In fact, more studies will likely be performed in the future. Although the relationship between birth weight and BP was not found in this generation in our study [55], it is not clear whether this generation or the subsequent generation will show similar patterns. A retrospective study is being performed to clarify this point, and a prospective study is also ongoing.

Proposed explanations for the relationship between birth weight and BP include inadequate maternal nutrition [59, 60], possibly leading to the acquisition of a reduced number of nephrons [61] and a reduced filtration area per glomerulus. This sequence of events applies a load to the kidney with growth and causes hypertension [62]. Since kidney formation occurs in the last 6–8 weeks of the gestational period, the growth of the nephron is also restricted [63] if growth impairment starts during this period. Moreover, it is established that the number of nephrons will not increase after birth [64], and autopsy studies have demonstrated a reduced number of nephrons in patients with essential hypertension [65, 66]. Furthermore, it has been reported that birth weight is linearly correlated with the number of nephrons [67] and that kidneys with reduced numbers of nephrons probably have a reduced estimated glomerular filtration rate (eGFR) [68]. Thus, birth weight may be a key determinant of eGFR in the young [69–71].

We recently investigated birth weight and eGFR changes in healthy Japanese adolescents (15- to 16-year olds) from 1998 to 2015 [72]. We also examined birth weight category trends to determine the contribution of birth weight changes on renal dysfunction rates. Our study found that the mean eGFR decreased from 105.1 ± 15.9 to 97.4 ± 13.8 mL/min/1.73 m^2^ and that the prevalence of mildly reduced renal function (eGFR ≤ 60– < 90 mL/min/1.73 m^2^) increased from 16.4% (1998–2003) to 30.0% (2010–2015) with simultaneous decreases in birth weight (from 3.21 ± 0.38 to 3.12 ± 0.38 kg) in our study population [72]. However, renal function was not measured directly by infusion of exogenous substances, such as inulin, in our study; instead, an equation for eGFR for Japanese adolescents with chronic kidney diseases was used [73]. Although this equation has not been validated in the general population [74] and may become less accurate at higher GFR levels, the equation was determined in individuals with GFR < 150 mL/min/1.73 m^2^ [73]. We also found that these outcome trends varied by birth weight category. The increased prevalence of mildly reduced renal function was particularly notable among the low-birth-weight participants. Multiple regression analyses demonstrated that low birth weight remained independently associated with mildly reduced renal function. Proteinuria also increased significantly over time, implying an increase in the renal dysfunction burden in the subject population. Thus, this study demonstrated the impact of the serial increase in low birth weight on renal function. Our findings may have implications for the broader Japanese population as well as for other populations in which the prevalence of low birth weight is increasing.

Other potential explanations for the relationship between birth weight and BP include some reports noting overexposure to maternal glucocorticoid levels in the womb [75, 76] or development disorders of blood vessels [77].

Low birth weight is a worldwide public health concern, demonstrating an increasing incidence in developed countries, including Japan. Low birth weight is also a risk factor for the subsequent development of chronic kidney disease. To date, studies have not evaluated the population impacts of increasing low birth weight rates on renal function. In a population of Japanese adolescents, the frequency of mildly reduced renal function increased as the frequency of low birth weight increased [72].

Other data have indicated that early childhood growth may be more important than birth weight as an influence on future BP. Children who were small at birth but had accelerated weight gain either very early after birth [78] or between the ages of 1 and 5 [46] had more insulin resistance, obesity, and hypertension later in life. This association between rapid postnatal weight gain and higher BP has been prospectively documented in 3-year olds [79], 8-year olds [80], and 11- to 14-year olds [81]. The authors also agree with this hypothesis. When a child born with a low birth weight subsequently becomes overweight, the risk of lifestyle-related diseases, such as hypertension, may increase beyond that of a child born at normal birth weight with approximately the same weight. In this context, a retrospective/prospective study is currently underway.

## BP tracking

A major problem with essential hypertension in children and adolescents is its development into adult essential hypertension. According to the results of a comparison study of BP at junior high school age and after 20 years in Japan, 20.9% of hypertensive junior high school students were still hypertensive after 20 years, whereas only 5.5% of normotensive individuals became hypertensive [1]. In a study in which university students were re-examined after 8–26 years, hypertension was observed in 44.6% of the hypertensive group but in only 9.2% of the normotensive group [2]. Conversely, in a large-scale study in the United States that followed 1505 children aged 5–14 years for 15 years or longer (Bogalusa Heart Study) [3], individuals who had elevated BP (>80th percentile) in childhood tended to have elevated BP 15 years later, 40% ranked by SBP and 37% ranked by DBP. The prevalence of hypertension in adulthood was much higher in individuals whose childhood BP was in the top quintile: 3.6 times higher (18% vs. 5%) for SBP and 2.6 times higher (15% vs. 5.8%) for DBP than individuals in every other quintile. More recently, data from the Fels Longitudinal study have added further weight to the concept that an elevated childhood BP reading predicts an increased chance of adult hypertension [4]. Such tracking is more consistent if the elevated childhood BP levels are combined with obesity, a parental history of hypertension, or increased left ventricular mass in echocardiography [82, 83]. However, it has been reported that the strength of tracking appears to decrease with longer periods of follow-up [84, 85]. Taken together, these data indicate that BP is predicted over time at a population level and supports interventions designed to prevent the future development of hypertension.

Since few follow-up studies have been conducted in students from high school to university in Japan, we previously evaluated the determinants of elevated BP in adolescents [86]. Specifically, we retrospectively evaluated the BP and anthropometric data in Japanese students during high school and university. We targeted male university students (mean age 21 years) who entered our university after graduating from our associated high school. Male university students who showed a BP above “high-normal” (SBP 130–139 mm Hg or DBP 85–89 mm Hg) at the age of 21 years exhibited a significantly higher BP, heart rate, body weight, and BMI than the normotensive participants when they were high school students. Stepwise regression analysis of the data showed that the best predictors of BP for male students at the age of 21 years were the initial high school BP and BMI levels and changes in BMI and heart rate during the 6-year follow-up period (Table 1). These results indicate that BP and BMI during high school and the changes in BMI and heart rate from high school to university influenced the BP at age 21 years in male students. These data also indicated that information on the prevention and management of hypertension, including weight control, should begin early, especially for male adolescents.

**Table 1:** Standardized regression coefficients and coefficients of determination in the stepwise linear regression models for predicting SBP and DBP at the age of 21 years from variables measured at the age of 15 years and their changes over the 6 years in male students [86]

Independent variable	Standardized regression coefficient
SBP at the age of 21 years	
SBP (15 years)	0.489
BMI (21 years) − BMI (15 years)	0.331
BMI (15 years)	0.237
HR (21 years) − HR (15 years)	0.185
Coefficient of determination = 0.374	
DBP at the age of 21 years	
HR (21 years) − HR (15 years)	0.396
BMI (21 years) − BMI (15 years)	0.277
SBP (15 years)	0.270
DBP (15 years)	0.198
HR (15 years)	0.186
BMI (15 years)	0.159
Coefficient of determination = 0.363	

*SBP* systolic blood pressure, *DBP* diastolic blood pressure, *BMI* body mass index, *HR* heart rate

Possible changes in lifestyle upon university entry may play a role in the above results. First, the time spent exercising may decrease. Second, some students may start to smoke and drink alcohol. Third, the frequency of eating outside the home is probably increased, and consequently, the subject’s salt intake may be increased. A lack of eating breakfast is another dietary concern. Lastly, an increase in stress originating from job concerns may be relevant [87]. In consideration of the above factors, initial interventions should be focused on lifestyle modifications. Nonpharmacological treatment involving therapeutic lifestyle changes and health-related behaviors is highly recommended in children and adolescents with essential hypertension in the early stage. Furthermore, male high school students with a heavier body weight and a higher BP should be regularly monitored for body weight and BP.

## Usefulness of regular exercise

The reduction of physical activity in adults in industrialized countries is becoming a serious concern with respect to lifestyle-related diseases such as hypertension [88], diabetes mellitus, and dyslipidemia. Although the decrease in exercise seems to be less detrimental to children and adolescents than to adults, these problems can affect even the younger generation [89]. Therefore, if the decrease in exercise in this generation negatively influences individual BP levels, steps must be taken to correct the lifestyles of such subjects.

Although the magnitude of change in BP may be modest, aerobic exercise, weight loss, and dietary modifications have been shown to reduce BP in children and adolescents [90–92]. For example, sustained training over 3–6 months has been shown to result in a reduction of 6–12 mm Hg for SBP and 3–5 mm Hg for DBP [93]. Exercise has been shown to improve body composition and reduce other cardiovascular risk factors associated with obesity [94]. However, the cessation of training is generally promptly followed by a rise in BP to pre-exercise levels. It is important to emphasize that aerobic exercise activities such as running, walking, and cycling are preferred to static forms of exercise in the management of hypertension.

Many reports have demonstrated the usefulness of regular exercise in preventing hypertension or as initial therapy for hypertension. The seventh [95] and eighth [96] reports of the Joint National Committee have also recommended regular exercise. Therefore, to investigate the usefulness of regular exercise during adolescence, we previously evaluated differences in BP and cardiovascular risk factors, including obesity, between male adolescents who belonged to sports clubs (exercise group) and male subjects of the same age group who did not belong to sports clubs (nonexercise group) [97]. The clinical characteristics of the two groups are shown in Table 2. We found that the exercise group had a lower percentage of body fat, a lower DBP value, and a lower heart rate, as well as a higher concentration of high-density lipoprotein (HDL) cholesterol and a lower concentration of triglycerides (TG) than the nonexercise group had (Table 2). Although the SBP in the exercise group tended to be lower than that of the nonexercise group, the difference was not statistically significant. However, upon admission to high school (age 15–16 years), the SBP, DBP, or heart rate in the exercise group was similar to that in the nonexercise group.

**Table 2:** Differences in blood pressure and cardiovascular risk factors between male adolescents (age range: 17–18 years) who belonged to sports clubs (exercise group) and male subjects of the same age group who did not belong to sports clubs (nonexercise group) [97]

	Exercise group	Nonexercise group
	*n* = 150	*n* = 114
Height (cm)	172.4 ± 5.5*	170.7 ± 5.0
Body weight (kg)	64.5 ± 7.7**	60.3 ± 8.2
Body mass index (kg/m^2^)	21.7 ± 2.3**	20.7 ± 2.6
Body fat (%)	13.6 ± 3.4**	14.9 ± 3.8
Systolic blood pressure (mm Hg)	118 ± 10	120 ± 12
Diastolic blood pressure (mm Hg)	64 ± 7*	66 ± 8
Mean blood pressure (mm Hg)	82 ± 7*	84 ± 9
Heart rate (/min)	70 ± 11**	76 ± 14
Total cholesterol (mg/dL)	164 ± 28	163 ± 25
HDL cholesterol (mg/dL)	63 ± 12*	60 ± 13
Triglycerides (mg/dL)	55 ± 23*	63 ± 27
Uric acid (mg/dL)	5.4 ± 1.0	5.5 ± 1.0
Fasting glucose (mg/dL)	88 ± 6	87 ± 6
Fasting insulin (μU/mL)	7.6 ± 2.6	7.7 ± 2.2
HOMA index	1.67 ± 0.64	1.66 ± 0.49
Positive FH of hypertension	33 (22.0%)	32 (28.1%)
Positive FH of diabetes mellitus	19 (12.7%)	18 (15.8%)

Values are mean ± SD. HOMA index = fasting glucose × fasting insulin/405

*HDL* high-density lipoprotein, *HOMA* homeostasis model assessment, *FH* family history

*p < 0.05, **p < 0.01 vs. nonexercise group

The types of sports have been classified into nine subgroups based on peak dynamic and static components during competition, as reported at the XXVI Bethesda Conference [98]. Thus, we divided the exercise group into three subgroups, which were classified according to the type and intensity of exercise. No differences in insulin resistance were found between the exercise group and the nonexercise group; however, a significant difference was found between students who preferred dynamic exercise and students in the nonexercise group (Tables 2 and 3). Namely, the students who preferred dynamic exercise exhibited lower BP and TG and a lower homeostasis model assessment (HOMA) index than did both students who did not belong to sports clubs and those who preferred less dynamic exercise in our study (Tables 2 and 3) [97]. Furthermore, a higher HOMA index was observed in students who preferred less dynamic exercise than in the students who did not belong to a sports club (Tables 2 and 3).

**Table 3:** Differences in blood pressure, lipid data, and HOMA index among the three subgroups, which are classified according to the type and intensity of exercise [97]

	Less dynamic exercise	Moderately dynamic exercise	Highly dynamic exercise
	*n* = 26	*n* = 62	*n* = 62
Height (cm)	172.3 ± 5.1	172.8 ± 5.6	171.9 ± 5.5
Body weight (kg)	66.1 ± 8.4	65.4 ± 8.5	62.9 ± 6.4
Body mass index (kg/m^2^)	22.3 ± 2.7	21.9 ± 2.6	21.3 ± 1.6
Body fat (%)	14.8 ± 4.2	13.8 ± 3.6	12.8 ± 2.7*
Systolic blood pressure (mm Hg)	119 ± 10	120 ± 11	116 ± 10^†^
Diastolic blood pressure (mm Hg)	65 ± 6	65 ± 7	63 ± 7
Mean blood pressure (mm Hg)	83 ± 7	83 ± 7	81 ± 8
Heart rate (/min)	75 ± 12	69 ± 11*	68 ± 11**
Total cholesterol (mg/dL)	168 ± 22	161 ± 24	165 ± 34
HDL cholesterol (mg/dL)	60 ± 15	64 ± 12	64 ± 12
Triglycerides (mg/dL)	66 ± 29	54 ± 22*	52 ± 21**
Fasting glucose (mg/dL)	88 ± 5	89 ± 8	88 ± 5
Fasting insulin (μU/mL)	9.1 ± 2.0	7.6 ± 3.0*	6.9 ± 2.0**
HOMA index	1.98 ± 0.43	1.70 ± 0.81	1.50 ± 0.46**

Values are mean ± SD. HOMA index = fasting glucose × fasting insulin/405

*HDL* high-density lipoprotein, *HOMA* homeostasis model assessment

*p < 0.05, **p < 0.01 vs. less dynamic exercise, ^†^*p* < 0.05 vs. moderately dynamic exercise

It should be noted that the terms “dynamic” and “static” exercise characterize activity based on mechanical action; thus, these terms differ from “aerobic” and “anaerobic” exercise. Nevertheless, high-intensity dynamic exercise that lasts for more than several minutes is considered to be performed primarily aerobically. Clearly, the advantages of aerobic exercise should be encouraged. In addition, it should be noted that aerobic exercise improves insulin resistance. Therefore, aerobic exercise should be recommended, even in teenagers, to help reduce the risk factors for cardiovascular disease, including hypertension.

Guideline-issuing organizations emphasize that the treatment of hypertension in children and adolescents should begin with nonpharmacological measures [7, 99]. As a nonpharmacological intervention, a study reported that moderate to intense aerobic exercise practiced for 40 min on 3–5 days a week improved vascular function, thereby reducing BP [92]. Furthermore, for the correction of obesity, exercise for pleasure is recommended. The total amount of daily exercise is important for the prevention of increases in BP regardless of the presence or absence of obesity [100].

## Questionnaire results about lifestyle

To further clarify the features of young hypertension and to identify preventive measures, we recently performed the following study [101]. Specifically, the differences between a hypertensive and normotensive group of university students and faculty and staff below 40 years of age were examined using medical checkup results and questionnaire results about lifestyle [101]. For university students, both men and women in the hypertensive group had significantly greater body weight and BMI than did those in the normotensive group and showed a significantly greater prevalence of obesity (BMI ≥ 25) (male hypertensive group, 23.4% of freshman and 17.1% of juniors; male normotensive group, 6.4% of freshman and 6.5% of juniors; female hypertensive group, 9.3% of freshman and 10% of juniors; female normotensive group, 1.9% of freshman and 2.6% of juniors). The results were similar to those previously obtained among male high school students [86] and in a recent study of female high school students (Takeda A, unpublished data). Moreover, in a previous study performed in university students [102], it was suggested that there was a close relationship between changes in weight and BP for 3 years, regardless of the original weight. Therefore, it seemed necessary not only for an obese person but also for a normal-weight person or a slightly obese person to be educated and guided on the importance of weight control during university enrollment as a measure to reduce the incidence of hypertension among students.

With regard to young obese individuals, it has been reported recently that severe obesity in children and young adults was associated with an increased prevalence of cardiometabolic risk factors, particularly among boys and young men [103]. That is, the study showed that the greater the severity of obesity was the higher the risks of low HDL cholesterol levels, high SBP and DBP, and high TG and glycated hemoglobin levels. In our study, although blood tests in this generation were not conducted, it was suggested that the early correction of obesity is important.

An increase in the factors associated with obesity in these university students, including lifestyle changes such as the initiation of drinking, was considered. Thus, we examined results from a questionnaire about drinking. Although differences were not observed between hypertensive and normotensive groups for both male and female freshmen, there were significantly more students who drank in the male hypertensive group among junior students (hypertensive group 5.0% vs. normotensive group 2.5%). Recently, an interesting report was published about drinking habits in young adults [104]. According to the report, compared to nonbinge drinkers, SBP at age 24 was 2.6 mm Hg higher among current monthly bingers (≥5 drinks/occasion) and 4.0 mm Hg higher among current weekly bingers. In addition, SBP at age 24 was 2.9 mm Hg higher among monthly bingers at age 20 and 3.6 mm Hg higher among weekly bingers at age 20. That is, frequent binge drinking at ages 20 and 24 was associated with higher SBP at age 24, which may be implicated in the development of hypertension. Moreover, the authors previously reported that 85% of the young adults who binged at age 20 sustained that behavior until at least age 24 [105]. It has also been reported that drinking patterns in early adulthood were strong predictors of both the frequency of alcohol use and problem drinking at ages 30 and 48 [106]. Thus, it appeared that education and instruction from an early age, before drinking habits begin, are required. In addition, there was no evidence for a significant association with changes in smoking, exercise, and eating habits other than drinking behavior in both male and female university students.

In faculty and staff below the age of 40 years, the body weight and BMI of the male hypertensive group were significantly higher than those of the male normotensive group. Moreover, there were significantly more obese persons (BMI ≥ 25) in the male hypertensive group (38.4% of the hypertensive group vs. 12.0% of the normotensive group) as well as in the group of university students. A significant difference between the hypertensive and normotensive groups was also identified in terms of abdominal circumference, fasting glucose, TG, and HDL cholesterol levels associated with a metabolic syndrome. In addition, a difference was also observed in the heart rate, uric acid, and low-density lipoprotein cholesterol levels (Table 4). Conversely, women showed patterns similar to those of men for these variables. Since there were few women in the hypertensive group, a significant difference could not be determined in part (Table 4). With regard to the relationship between BP and obesity observed in faculty and staff below the age of 40, a reference to prior education also emerged to some extent from the questionnaire results. That is, regarding the item that showed a significant difference between the male hypertensive and normotensive group, we found significantly more “yes” responses to the question “Is your present body weight 10 kg or more than your body weight at age 20 years?” in the male hypertensive group (hypertensive group 32.2% vs. normotensive group 14.9%). Again, since there were few women in the hypertensive group, a significant difference could not be determined, but women showed similar tendencies (hypertensive group 10.2% vs. normotensive group 4.6%, *p* = 0.07). Although this generation has still differed only approximately 10 years from the 20-year olds, for the person whose body weight increased by 10 kg or more, it became clear that the BP undoubtedly tended to increase. As mentioned above, body weight is a major influence on the BP of this generation. Therefore, as we have previously reported for university students [102], sufficient attention should be paid to obvious increases in body weight in individuals in their 20s regardless of their present body weight, and the necessity of prior education and instruction has been suggested. Moreover, only in the male hypertensive group of this generation were there significantly more smokers (hypertensive group 15.7% vs. normotensive group 10.1%) and more respondents who answered that they “often drink” (hypertensive group 20.1% vs. normotensive group 11.7%). Therefore, it is also necessary to pay particular attention to this point at the time of lifestyle instruction for individuals with higher BP. In addition, there were no significant group or sex differences in the content of the questionnaire relative to other lifestyle habits, such as exercise habits, sleep patterns, and stress, among others.

**Table 4:** Comparison between normotensive and hypertensive groups among faculty and  staff below age 40 years [101]

	Male			Female		
	Normotensive group	Hypertensive group	*p* Value	Normotensive group	Hypertensive group	*p* Value
	*n* = 920	*n* = 203		*n* = 1674	*n* = 49	
Age (years)	32.0 ± 4.0	32.7 ± 4.3	0.02	29.7 ± 4.9	32.0 ± 5.5	<0.01
Height (cm)	172.2 ± 5.7	172.8 ± 6.7	0.3	159.2 ± 5.2	158.8 ± 5.4	0.58
Body weight (kg)	65.2 ± 2.6	73.0 ± 12.0	<0.01	50.9 ± 6.8	56.6 ± 11.7	<0.01
BMI (kg/m^2^)	21.9 ± 2.6	24.5 ± 3.8	<0.01	20.1 ± 2.4	22.4 ± 4.3	<0.01
Abdominal circumference (cm)	78.2 ± 7.7	84.9 ± 10.4	<0.01	71.4 ± 6.8	76.3 ± 10.5	<0.01
Systolic blood pressure (mm Hg)	114 ± 9	140 ± 10	<0.01	104 ± 10	137 ± 10	<0.01
Diastolic blood pressure (mm Hg)	68 ± 7	83 ± 8	<0.01	62 ± 8	82 ± 9	<0.01
Heart rate (/min)	73 ± 11	79 ± 13	<0.01	75 ± 11	88 ± 13	<0.01
Creatinine (mg/dL)	0.86 ± 0.10	0.86 ± 0.11	0.64	0.63 ± 0.09	0.64 ± 0.09	0.52
Uric acid (mg/dL)	6.1 ± 1.2	6.6 ± 1.3	<0.01	4.4 ± 0.9	4.7 ± 1.1	0.04
Fasting glucose (mg/dL)	98 ± 11	103 ± 13	<0.01	94 ± 10	98 ± 9	0.02
Triglycerides (mg/dL)	95 ± 74	136 ± 106	<0.01	66 ± 38	73 ± 36	0.16
HDL cholesterol (mg/dL)	59 ± 13	55 ± 13	<0.01	69 ± 13	65 ± 16	0.11
LDL cholesterol (mg/dL)	112 ± 28	124 ± 31	<0.01	98 ± 25	107 ± 30	<0.01

Values are mean ± SD

*BMI* body mass index, *HDL* high-density lipoprotein, *LDL* low-density lipoprotein

## Conclusion

Considering the existence of BP tracking phenomenon, early education and instruction (especially regarding weight control and recommendations for exercise) are necessary, especially for male high school students who have a moderately high BP and show a tendency toward obesity, even in this generation. Moreover, it appears that students with a positive family history of hypertension and those who are born with a low birth weight require the same preventive measures. Since significant lifestyle changes, such as the initiation of drinking alcohol, will undoubtedly occur when students begin university, opportunities for early education and instruction about these habits and their association with BP are required. In particular, for male students with higher BP during high school, it appears that these precautions regarding an increase in body weight are required irrespective of the degree of obesity. For young adults below the age of 40, the importance of body weight and its association with hypertension requires proper education and instruction. Concerning lifestyle habits, special precautions regarding drinking and smoking in male hypertensives are required. Thus, hypertension at a young age also has a strong association with obesity. It has previously been reported in the United States [107] and Japan that there are significantly more hypertensive patients among obese individuals than among nonobese individuals, and the prevalence of hypertension increases as the degree of obesity increases. Although these young obese individuals may already have a lifestyle-related disease, suitable management is necessary so that their BP does not lead to adults. Therefore, both BP measurement and the management of obesity are considered to be efficient methods for the detection and medical treatment of young hypertension. For the lifetime prevention of hypertension, it is very important to be aware of one’s own health condition and to learn proper lifestyle habits beginning in childhood. There have been recent reports indicating that individuals with stage 1 and 2 hypertension before the age of 40, as defined by the BP classification in the 2017 American College of Cardiology/American Heart Association guidelines [108], showed a significantly higher risk of subsequent cardiovascular disease events than those with normal BP before the age of 40 [109, 110]. BP measurement should be an objective for the lifetime prevention of hypertension. BP measurement and BP classification may help identify young adults at higher risk for cardiovascular disease events.

Finally, with the inclusion of the reports of other researchers, we summarized the main factors that are currently thought to be involved in BP levels in children and adolescents (Table 5).

**Table 5:** Factors contributing to blood pressure levels in children and adolescents

Genetic	Parental and sibling blood pressure levels
	Increased salt sensitivity
	Autonomic abnormalities
	Obesity
Environmental	Exercise
	Birth weight
	Socioeconomic status
	Neonatal weight gain
	Early childhood growth
Mixed genetic and environmental	Height
	Sexual maturation
	Sympathetic nervous system reactivity
	Pulse rate
	Sodium and other nutrient intakes
	Stress
	Body mass
	Weight

## References

[1] Uchiyama M (1994). Risk factors for the development of essential hypertension: long-term follow-up study in junior high school students in Niigata, Japan. J Hum Hypertens.

[2] Kawasaki T, Uezono K, Sanefuji M, Utsunomiya H, Fujino T, Kanaya S (2003). A 17-year follow-up study of hypertensive and normotensive male university students in Japan. Hypertens Res.

[3] Bao W, Threefoot SA, Srinivasan SR, Berenson GS (1995). Essential hypertension predicted by tracking of elevated blood pressure from childhood to adulthood: the Bogalusa Heart Study. Am J Hypertens.

[4] Carrico RJ, Sun SS, Sima AP, Rosner B (2013). The predictive value of childhood blood pressure values for adult elevated blood pressure. Open J Pediatr.

[5] Shimamoto K, Ando K, Fujita T, Hasebe N, Higaki J, Horiuchi M (2014). Japanese Society of Hypertension Committee for Guidelines for the Management of Hypertension. The Japanese Society of Hypertension Guidelines for the Management of Hypertension (JSH 2014). Hypertens Res.

[6] Flynn JT, Kaelber DC, Baker-Smith CM, Blowey D, Carroll AE, Daniels SR (2017). Clinical practice guideline for screening and management of high blood pressure in children and adolescents. Pediatrics.

[7] National High Blood Pressure Education Program Working Group on High Blood Pressure in Children and Adolescents. (2004). The fourth report on the diagnosis, evaluation, and treatment of high blood pressure in children and adolescents. Pediatrics.

[8] Uchiyama M, Sakai K (1985). Studies of blood pressures in school children in northern Japan. Public Health.

[9] Tochikubo O, Sasaki O, Umemura S, Kaneko Y (1986). Management of hypertension in high school students by using new salt titrator tape. Hypertension.

[10] McNiece KL, Gupta-Malhotra M, Samuels J, Bell C, Garcia K, Poffenbarger T (2007). Left ventricular hypertrophy in hypertensive adolescents. Analysis of risk by 2004 National High Blood Pressure Education Program Working Group staging criteria. Hypertension.

[11] Sorof JM, Poffenbarger T, Franco K, Bernard L, Portman RJ (2002). Isolated systolic hypertension, obesity, and hyperkinetic hemodynamic states in children. J Pediatr.

[12] Sorof JM, Lai D, Turner J, Poffenbarger T, Portman RJ (2004). Overweight, ethnicity, and the prevalence of hypertension in school-aged children. Pediatrics.

[13] Din-Dzietham R, Liu Y, Bielo MV, Shamsa F (2007). High blood pressure trends in children and adolescents in national surveys, 1963 to 2002. Circulation.

[14] Cao ZQ, Zhu L, Zhang T, Wu L, Wang Y (2012). Blood pressure and obesity among adolescents: a school-based population study in China. Am J Hypertens.

[15] Steinhorsdottir SD, Eliasdottir SB, Indridason OS, Agustsdottir IM, Palsson R, Edvardsson VO (2011). Prevalence of hypertension in 9- to 10-year-old Icelandic school children. J Clin Hypertens.

[16] Flynn J (2013). The changing face of pediatric hypertension in the era of the childhood obesity epidemic. Pediatr Nephrol.

[17] Wühl E, Witte K, Soergel M, Mehls O, Schaefer F (2002). Distribution of 24-h ambulatory blood pressure in children: normalized reference values and role of body dimensions. J Hypertens.

[18] Stergiou GS, Yiannes NG, Rarra VC, Panagiotakos DB (2007). Home blood pressure normalcy in children and adolescents: the Arsakeion School study. J Hypertens.

[19] Stergiou GS, Ntineri A, Kollias A, Stambolliu E, Kapogiannis A, Vazeou A (2018). Home blood pressure monitoring in pediatric hypertension: the US perspective and a plan for action. Hypertens Res.

[20] Sun J, Steffen LM, Ma C, Liang Y, Xi B (2017). Definition of pediatric hypertension: are blood pressure measurements on three separate occasions necessary?. Hypertens Res.

[21] Biron P, Mongeau JG, Bertrand D (1976). Familial aggregation of blood pressure in 558 adopted children. Can Med Assoc J.

[22] Miall W. E., Oldham P. D. (1963). The Hereditary Factor in Arterial Blood-pressure. BMJ.

[23] Havlik RJ, Garrison RJ, Feinleib M, Kannel WB, Castelli WP, Mcnamara M (1979). Blood pressure aggregation in families. Am J Epidemiol.

[24] Flynn JT, Alderman MH (2005). Characteristics of children with primary hypertension seen at a referral center. Pediatr Nephrol.

[25] Robinson RF, Batisky DL, Hayes JR, Nahata MC, Mahan JD (2005). Significance of heritability in primary and secondary pediatric hypertension. Am J Hypertens.

[26] Wang X, Wang B, Chen C, Yang J, Fang Z, Zuckerman B (1999). Familial aggregation of blood pressure in a rural Chinese community. Am J Epidemiol.

[27] Kupper N, Ge D, Treiber FA, Snieder H (2006). Emergence of novel genetic effects on blood pressure and hemodynamics in adolescence. The Georgia Cardiovascular Twin Study. Hypertension.

[28] Hunt SC, Williams RR, Barlow GK (1986). A comparison of positive family history definitions for defining risk of future disease. J Chronic Dis.

[29] Kawabe H, Saito I, Nagano S, Saruta T (1994). Relation of home blood pressure to body weight in young normotensive men with or without family history of hypertension. Am J Hypertens.

[30] Fagard RH, Van Den Broeke C, De Cort P (2005). Prognostic significance of blood pressure measured in the office, at home and during ambulatory monitoring in older patients in general practice. J Hum Hypertens.

[31] Sega R, Facchetti R, Bombelli M, Cesana G, Corrao G, Grassi G (2005). Prognostic value of ambulatory and home blood pressures compared with office blood pressure in the general population. Follow-up results from the Pressioni Arteriose Monitorate e Loro Associazioni (PAMELA) study. Circulation.

[32] Staessen JA, Thijs L, Fagard R, O’Brien ET, Clement D, de Leeuw PW (1999). for the Systolic Hypertension in Europe Trial Investigators. Predicting cardiovascular risk using conventional vs ambulatory blood pressure in older patients with systolic hypertension. J Am Med Assoc.

[33] Mossberg HO (1989). 40-year follow-up of overweight children. Lancet.

[34] Kotsis V, Stabouli S, Papakatsika S, Rizos Z, Parati G (2010). Mechanisms of obesity-induced hypertension. Hypertens Res.

[35] Wirix AJ, Kaspers PJ, Nauta J, Chinapaw MJM, Kist-van Holthe JE (2015). Pathophysiology of hypertension in obese children: a systematic review. Obes Rev.

[36] Hall JE, do Carmo JM, da Silva AA, Wang Z, Hall ME (2015). Obesity-induced hypertension. Interaction of neurohumoral and renal mechanisms. Circ Res.

[37] Leggio M, Lombardi M, Caldarone E, Severi P, D’Emidio S, Armeni M (2017). The relationship between obesity and hypertension: an updated comprehensive overview on vicious twins. Hypertens Res.

[38] Ministry of Health, Labour and Welfare. Vital statistics in Japan. 2018; http://www.mhlw.go.jp/english/database/db-hw/vs01.html. Accessed 23 Nov 2018.

[39] Organization for Economic Cooperation and Development. OECD Family Database. 2018; https://www.oecd.org/els/family/CO_1_3_Low_birth_weight.pdf. Accessed 23 Nov 2018.

[40] Oken E (2013). Secular trends in birthweight. Nestle Nutr Inst Workshop Ser.

[41] Tsukamoto H, Fukuoka H, Koyasu M, Nagai Y, Takimoto H (2007). Risk factors for small for gestational age. Pediatr Int.

[42] Gennser G, Rymark P, Isberg PE (1988). Low birth weight and risk of high blood pressure in adulthood. Br Med J.

[43] Barker DJP (1996). The fetal origins of hypertension. J Hypertens.

[44] Law CM, Shiell AW (1996). Is blood pressure inversely related to birth weight? The strength of evidence from a systematic review of the literature. J Hypertens.

[45] Gamborg M, Byberg L, Rasmussen F, Andersen PK, Baker JL, Bengtsson C (2007). Birth weight and systolic blood pressure in adolescence and adulthood: meta-regression analysis of sex- and age-specific results from 20 Nordic studies. Am J Epidemiol.

[46] Law CM, Shiell AW, Newsome CA, Syddall HE, Shinebourne EA, Fayers PM (2002). Fetal, infant, and childhood growth and adult blood pressure. A longitudinal study from birth to 22 years of age. Circulation.

[47] Zureik M, Bonithon-Kopp C, Lecomte E, Siest G, Ducimetiere P (1996). Weights at birth and in early infancy, systolic pressure, and left ventricular structure in subjects aged 8 to 24 years. Hypertension.

[48] Higgins M, Keller J, Moore F, Ostrander L, Metzner H, Stock L (1980). Studies of blood pressure in Tecumseh, Michigan. Am J Epidemiol.

[49] Paneth N, Ahmed F, Stein AD (1996). Early nutritional origins of hypertension: a hypothesis still lacking support. J Hypertens.

[50] Falkner B, Hulman S, Katz S, Kushner H (1997). Birth weight is not associated with adult blood pressure in African Americans. Am J Hypertens.

[51] Falkner B, Kushner H, Katz S (1996). Birth weight, childhood growth, and adult blood pressure in African Americans. J Am Soc Nephrol.

[52] Hashimoto N, Kawasaki T, Kikuchi T, Takahashi H, Uchiyama M (1996). The relationship between the intrauterine environment and blood pressure in 3-year-old Japanese children. Acta Paediatr.

[53] Miura K, Nakagawa H, Tabata M, Morikawa Y, Nishijo M, Kagamimori S (2001). Birth weight, childhood growth, and cardiovascular disease risk factors in Japanese aged 20 years. Am J Epidemiol.

[54] Law C (1995). Fetal influences on adult hypertension. J Hum Hypertens.

[55] Kawabe H, Shibata H, Hirose H, Tsujioka M, Saito I, Saruta T (1999). Sexual differences in relationships between birth weight or current body weight and blood pressure or cholesterol in young Japanese students. Hypertens Res.

[56] Siewert-Delle A, Ljungman S (1998). The impact of birth weight and gestational age on blood pressure in adult life. A population-based study of 49-year-old men. Am J Hypertens.

[57] Lauer RM, Clarke WR (1989). Childhood risk factors for high adult blood pressure: the Muscatine Study. Pediatrics.

[58] Holland FJ, Stark O, Ades AE, Peckham CS (1993). Birth weight and body mass index in childhood, adolescence, and adulthood as predictors of blood pressure at age 36. J Epidemiol Community Health.

[59] Barker DJ, Godfrey KM, Gluckman PD, Harding JE, Owens JA, Robinson JS (1993). Fetal nutrition and cardiovascular disease in adult life. Lancet.

[60] Law CM, Barker DJ, Bull AR, Osmond C (1991). Maternal and fetal influences on blood pressure. Arch Dis Child.

[61] Mackenzie HS, Lawler EV, Brenner BM (1996). Congenital oligonephropathy: the fetal flaw in essential hypertension?. Kidney Int.

[62] Mackenzie HS, Brenner BM (1995). Fewer nephrons at birth: a missing link in the etiology of essential hypertension?. Am J Kidney Dis.

[63] Hinchliffe SA, Lynch MRJ, Sargent PH, Howard CV, Van Velzen D (1992). The effect of intrauterine growth retardation on the development of renal nephrons. Br J Obstet Gynaecol.

[64] Lucas A, Morley R (1994). Does early nutrition in infants born before term programme later blood pressure?. Br Med J.

[65] Keller G, Zimmer G, Mall G, Ritz E, Amann K (2003). Nephron number in patients with primary hypertension. N Engl J Med.

[66] Brenner BM, Chertow GM (1994). Congenital oligonephropathy and the etiology of adult hypertension and progressive renal injury. Am J Kidney Dis.

[67] Hughson M, Farris AB, Douglas-Denton R, Hoy WE, Bertram JF (2003). Glomerular number and size in autopsy kidneys: the relationship to birth weight. Kidney Int.

[68] Luyckx VA, Bertram JF, Brenner BM, Fall C, Hoy WE, Ozanne SE (2013). Effect of fetal and child health on kidney development and long-term risk of hypertension and kidney disease. Lancet.

[69] Bakker H, Gaillard R, Franco OH, Hofman A, van der Heijden AJ, Steegers EAP (2014). Fetal and infant growth patterns and kidney function at school age. J Am Soc Nephrol.

[70] Keijzer-Veen MG, Kleinveld HA, Lequin MH, Dekker FW, Nauta J, de Rijke YB (2007). Renal function and size at young adult age after intrauterine growth restriction and very premature birth. Am J Kidney Dis.

[71] Hallan S, Euser AM, Irgens LM, Finken MJJ, Holmen J, Dekker FW (2008). Effect of intrauterine growth restriction on kidney function at young adult age: The Nord Trǿndelag Health (HUNT 2) Study. Am J Kidney Dis.

[72] Kanda T, Tekeda A, Hirose H, Abe T, Urai H, Inokuchi M (2018). Temporal trends in renal function and birthweight in Japanese adolescent males (1998-2015). Nephrol Dial Transplant.

[73] Uemura O, Nagai T, Ishikura K, Ito S, Hataya H, Gotoh Y (2014). Creatinine-based equation to estimate the glomerular filtration rate in Japanese children and adolescents with chronic kidney disease. Clin Exp Nephrol.

[74] Uemura O, Nagai T, Ishikura K, Ito S, Hataya H, Gotoh Y (2015). Reference glomerular filtration rate levels in Japanese children: using the creatinine and cystatin C based estimated glomerular filtration rate. Clin Exp Nephrol.

[75] Benediktsson R, Lindsay RS, Noble J, Seckl JR, Edwards CRW (1993). Glucocorticoid exposure in utero: new model for adult hypertension. Lancet.

[76] Seckl JR (1997). Glucocorticoids, feto-placental 11β-hydroxysteroid dehydrogenase type 2, and the early life origins of adult disease. Steroids.

[77] Chapman N, Mohamudally A, Stanton A, Sever P, Sayer AA, Cooper C (1996). Vascular network geometry—the missing link between birth weight and cardiovascular risk?. J Hypertens.

[78] Singhal A, Fewtrell M, Cole TJ, Lucas A (2003). Low nutrient intake and early growth for later insulin resistance in adolescents born preterm. Lancet.

[79] Belfort MB, Rifas-Shiman SL, Rich-Edwards J, Kleinman KP, Gillman MW (2007). Size at birth, infant growth, and blood pressure at three years of age. J Pediatr.

[80] Burke V, Beilin LJ, Blake KV, Doherty D, Kendall GE, Newnham JP (2004). Indicators of fetal growth do not independently predict blood pressure in 8-year-old Australians. A prospective cohort study. Hypertension.

[81] Falkner B, Hulman S, Kushner H (2004). Effect of birth weight on blood pressure and body size in early adolescence. Hypertension.

[82] Lauer RM, Clarke WR, Mahoney LT, Witt J (1993). Childhood predictors for high adult blood pressure. The Muscatine Study. Pediatr Clin North Am.

[83] Shear CL, Burke GL, Freedman DS, Berenson GS (1986). Value of childhood blood pressure measurements and family history in predicting future blood pressure status: results from 8 years of follow-up in the Bogalusa Heart Study. Pediatrics.

[84] Chen X, Wang Y (2008). Tracking of blood pressure from childhood to adulthood. A systematic review and meta-regression analysis. Circulation.

[85] Toschke AM, Kohl L, Mansmann U, von Kries R (2010). Meta-analysis of blood pressure tracking from childhood to adulthood and implications for the design of intervention trials. Acta Paediatr.

[86] Kawabe H, Shibata H, Hirose H, Tsujioka M, Saito I, Saruta T (2000). Determinants for the development of hypertension in adolescents. A 6-year follow-up. J Hypertens.

[87] Falkner B, Onesti G, Angelakos ET, Fernandes M, Langman C (1979). Cardiovascular response to mental stress in normal adolescents with hypertensive parents. Hemodynamic and mental stress in adolescents. Hypertension.

[88] Anderssen N, Jacobs DR, Sidney S, Bild DE, Stemfeld B, Stattery ML (1996). Change and secular trends in physical activity patterns in young adults: a seven-year longitudinal follow-up in the coronary artery risk development in young adults study (CARDIA). Am J Epidemiol.

[89] Shea S, Basch CE, Gutin B, Stein AD, Contento IR, Irigoyen M (1994). The rate of increase in blood pressure in children 5 years of age is related to changes in aerobic fitness and body mass index. Pediatrics.

[90] Bianchini JAA, da Silva DF, Nardo CCS, Carolino IDR, Hernandes F, Junior NN (2013). Multidisciplinary therapy reduces risk factors for metabolic syndrome in obese adolescents. Eur J Pediatr.

[91] Maggio ABR, Aggoun Y, Martin XE, Marchand LM, Beghetti M, Farport-Lambert NJ (2011). Long-term follow-up of cardiovascular risk factors after exercise training in obese children. Int J Pediatr.

[92] Torrance B, McGuire KA, Lewanczuk R, McGavock J (2007). Overweight, physical activity and high blood pressure in children: a review of the literature. Vasc Health Risk Manag.

[93] Alpert BS (2000). Exercise as a therapy to control hypertension in children. Int J Sports Med.

[94] Zorba E, Cengiz T, Karacabey K (2011). Exercise training improves body composition, blood lipid profile and serum insulin levels in obese children. J Sports Med Phys Fit.

[95] Chobanian AV, Bakris GL, Black HR, Cushman WC, Green LA, Izzo JL (2003). The Seventh Report of the Joint National Committee on Prevention, Detection, Evaluation, and Treatment of High Blood Pressure. The JNC 7 report. JAMA.

[96] James PA, Oparil S, Carter BL, Cushman WC, Dennison Himmelfarb C, Handler J (2014). 2014 evidence-based guideline for the management of high blood pressure in adults. Report from the panel members appointed to the Eighth Joint National Committee (JNC 8). JAMA.

[97] Kawabe H, Murata K, Shibata H, Hirose H, Tsujioka M, Saito I (2000). Participation in school clubs and related effects on cardiovascular risk factors in young males. Hypertens Res.

[98] Mitchell JH, Haskell WL, Raven PB (1994). Classification of sports. Med Sci Sports Exerc.

[99] Expert Panel on Integrated Guidelines for Cardiovascular Health and Risk Reduction in Children and Adolescents; National Heart, Lung, and Blood Institute. (2011). Expert panel on integrated guidelines for cardiovascular health and risk reduction in children and adolescents: summary report. Pediatrics.

[100] Leary SD, Ness AR, Smith GD, Mattocks C, Deere K, Blair SN (2008). Physical activity and blood pressure in childhood: findings from a population-based study. Hypertension.

[101] Kawabe H, Takeda A, Kanda T, Hirose H (2017). Comparison between hypertensives and normotensives in university students and young faculty stuff: evaluation of results in annual health checkups and lifestyle questionnaire (in Japanese). Bull Keio Univ Health Cent.

[102] Hirose H, Saito I, Tsujioka M, Kawabe H, Saruta T (2000). Effects of body weight control on changes in blood pressure: three-year follow-up study in young Japanese individuals. Hypertens Res.

[103] Skinner AC, Perrin EM, Moss LA, Skelton JA (2015). Cardiometabolic risks and severity of obesity in children and young adults. N Engl J Med.

[104] Wellman RJ, Vaughn JA, Sylvestre MP, O’Loughlin EK, Dugas EN, O’Loughlin JL (2016). Relationships between current and past binge drinking and systolic blood pressure in young adults. J Adolesc Health.

[105] Wellman RJ, Contreras GA, Dugas EN, O’Loughlin EK, O’Loughlin JL (2014). Determinants of sustained binge drinking in young adults. Alcohol Clin Exp Res.

[106] Zucker RA (2008). Anticipating problem alcohol use developmentally from childhood into middle adulthood: what have we learned?. Addiction.

[107] Friedemann C, Heneghan C, Mahtani K, Thompson M, Perera R, Ward AM (2012). Cardiovascular disease risk in healthy children and its association with body mass index: systematic review and meta-analysis. Br Med J.

[108] Whelton PK, Carey RM, Aronow WS, Casey DE, Collins KJ, Dennison Himmelfarb C (2018). 2017 ACC/AHA/AAPA/ABC/ACPM/AGS/APhA/ASH/ASPC/NMA/PCNA guideline for the prevention, detection, evaluation, and management of high blood pressure in adults: a report of the American College of Cardiology/American Heart Association Task Force on clinical practice guidelines. J Am Coll Cardiol.

[109] Yano Y, Reis JP, Colangelo LA, Shimbo D, Viera AJ, Allen NB (2018). Association of blood pressure classification in young adults using the 2017 American College of Cardiology/American Heart Association Blood Pressure Guideline with cardiovascular events later in life. J Am Med Assoc.

[110] Son JS, Choi S, Kim K, Kim SM, Choi D, Lee G (2018). Association of blood pressure classification in Korea young adults according to the 2017 American College of Cardiology/American Heart Association Guidelines with subsequent cardiovascular disease events. J Am Med Assoc.

